# The Effectiveness of Tobacco Marketing Regulations on Reducing Smokers’ Exposure to Advertising and Promotion: Findings from the International Tobacco Control (ITC) Four Country Survey

**DOI:** 10.3390/ijerph8020321

**Published:** 2011-01-26

**Authors:** Karin A. Kasza, Andrew J. Hyland, Abraham Brown, Mohammad Siahpush, Hua-Hie Yong, Ann D. McNeill, Lin Li, K. Michael Cummings

**Affiliations:** 1 Division of Cancer Prevention and Population Sciences, Roswell Park Cancer Institute, Elm and Carlton Streets, Buffalo, NY 14263, USA; 2 Department of Health Behavior, Roswell Park Cancer Institute, Elm and Carlton Streets, Buffalo, NY 14263, USA; E-Mails: andrew.hyland@roswellpark.org (A.J.H.); michael.cummings@roswellpark.org (K.M.C.); 3 Centre for Tobacco Control Research, Institute for Social Marketing, University of Stirling, Stirling FK9 4LA, Scotland, UK; E-Mail: akb2@stir.ac.uk; 4 Department of Health Promotion, Social and Behavioral Health, University of Nebraska Medical Center, 42nd and Emile Streets, Omaha, NE 68198, USA; E-Mail: msiahpush@unmc.edu; 5 VicHealth Centre for Tobacco Control, The Cancer Council Victoria, 100 Drummond Street, Carlton, Victoria 3053, Australia; E-Mails: hua.yong@cancervic.org.au (H.-H.Y.); lin.li@cancervic.org.au (L.L.); 6 Division of Epidemiology and Public Health, Nottingham City Hospital, University of Nottingham, Hucknall Road, Nottingham NG5 1PB, UK; E-Mail: ann.mcneill@nottingham.ac.uk

**Keywords:** tobacco control, marketing regulation, socioeconomic differences, public policy

## Abstract

Exposure to tobacco product marketing promotes the initiation, continuation, and reuptake of cigarette smoking and as a result the World Health Organization Framework Convention on Tobacco Control (WHO FCTC) has called upon member Parties to enact comprehensive bans on tobacco advertising and promotion. This study examines the immediate and long term effectiveness of advertising restrictions enacted in different countries on exposure to different forms of product marketing, and examines differences in exposure across different socioeconomic status (SES) groups. Nationally representative data from the United Kingdom, Canada, Australia, and the United States, collected from adult smokers between 2002 and 2008 using the International Tobacco Control Four Country Survey (ITC-4), were used in this study (N = 21,615). In light of the specific marketing regulation changes that occurred during the course of this study period, changes in awareness of tobacco marketing via various channels were assessed for each country, and for different SES groups within countries. Tobacco marketing regulations, once implemented, were associated with significant reductions in smokers’ reported awareness of pro-smoking cues, and the observed reductions were greatest immediately following the enactment of regulations. Changes in reported awareness were generally the same across different SES groups, although some exceptions were noted. While tobacco marketing regulations have been effective in reducing exposure to certain types of product marketing there still remain gaps, especially with regard to in-store marketing and price promotions.

## 1. Introduction

Marketing theory suggests several mechanisms through which tobacco advertising and promotion can be effective, including: increasing experimentation with/initiation of smoking, increasing individuals’ cigarette consumption, decreasing quit attempts/quit success rates, and enticing former smokers to begin smoking again [1]. Empirical research confirms the role that marketing plays in fostering positive smoking attitudes and expectations among adolescents which, in turn, increases their likelihood of becoming smokers [2,3]. Marketing efforts have also been successful in portraying smoking as being normal and socially acceptable to adults, making it less likely for current smokers to feel compelled to quit [4,5]. Further, the presence of tobacco advertisements in stores, particularly cigarette displays, makes it harder for smokers who try to quit smoking to be successful in doing so [6].

Given the harmful influence of tobacco advertising and promotion on current, potential, and former smokers, marketing regulation is a necessary component in the effort to combat the global tobacco epidemic. The World Health Organization Framework Convention on Tobacco Control (the WHO FCTC) has responded by calling on member Parties to implement extensive tobacco regulatory strategies, including the enactment of comprehensive bans on tobacco advertising, promotion, and sponsorship activities [7]. Such bans have been shown to be effective in reducing tobacco consumption, both in developed countries [8] and in developing countries [9].

Since 2002, various tobacco marketing regulations have been enacted in the United Kingdom (UK), Canada, Australia and the United States. Table 1 presents an overview of the extent of these marketing regulations in each of the four countries.

In the UK, the Tobacco Advertising and Promotion Act 2002 (TAPA) banned almost all types of tobacco marketing according to the following timeline: by 2003, print and broadcast media, billboard advertisements, arts sponsorships, and free sample, special price, gift/discount, leaflet, Internet, and email promotions were banned [10]. Sports sponsorships were partially banned in 2003 and completely banned by 2005. Complete regulation of store advertisements, direct mailings and sign promotions did not come into effect during the course of the study period. Since then, however, the Tobacco and Primary Medical Services (Scotland) Act 2010 was passed, and it placed further restrictions on the retailing of tobacco products [11]. In the UK, the Health Act was passed in 2009 which would require the removal of tobacco displays at point of sale and all cigarette vending machines, but this law has yet to go into effect.

The Tobacco Act 1997 banned various types of marketing in Canada, including movie and billboard advertisements, competitions, free samples and gift/discount promotions [12]. Sports and arts sponsorships were partially banned by the Act, and completely banned in 2003. Store advertisements were partially banned by the Act, and retail displays of cigarettes in stores were widely outlawed in 2008. Restrictions on electronic tobacco promotions, direct mailings, branded products, leaflets and signs remained partial throughout the course of the study period (in general, the Act banned lifestyle advertising but allowed for informational advertising when directed toward adults).

In Australia, the Tobacco Advertising Prohibition (TAP) Act 1992 outlawed most forms of tobacco marketing [13]. Exceptions included special price promotions and point-of-sale displays of cigarettes in stores. Free sample promotions were prohibited in some Australian states, and gift/discount promotions were restricted with some exemptions remaining in most states. Sports sponsorships were banned, but exceptions remained for Formula 1 Grand Prix motor racing until 2006.

The United States (US) has been slower to adopt tobacco marketing restrictions compared to the other countries. The Master Settlement Agreement (1998) placed some modest restrictions on tobacco product marketing, in particular, banning participating manufacturers from advertising on billboards larger than 14 square feet, and banning print advertising in magazines with a significant youth readership [14]. In 2009, the US Food and Drug Administration enacted “Regulations Restricting the Sale and Distribution of Cigarettes and Smokeless Tobacco to Protect Children and Adolescents,” which will further reduce the appeal of cigarettes to youth [15].

The effectiveness of these marketing bans in bringing about a favorable change in smoking behavior, however, depends on the extent to which bans produce a genuine reduction in actual exposure to tobacco marketing activities. Harris *et al.* (2006) evaluated the effects of the UK Tobacco Advertising and Promotion Act 2002 (TAPA) on smokers’ exposure to tobacco marketing, as measured by self-reported awareness of marketing activities, using data from the first two waves (2002, 2003) of the International Tobacco Control Four Country Survey (ITC-4) [16]. Harris found that change in awareness of different types of tobacco product marketing was much greater in the UK than in the comparison countries not affected by the ban (*i.e.*, Canada, Australia, and the United States), and that the greatest declines in awareness were among the channels where regulations came into force. These results demonstrate the effectiveness of TAPA in reducing tobacco marketing awareness among smokers in the UK immediately post-ban.

Using an additional five waves of data from the ITC-4 survey (total of 7 waves collected between 2002 and 2008), the current study extends these findings by evaluating the longer term impact of the UK ban on smokers’ awareness of tobacco marketing, along with the impact of other marketing bans enacted in other countries between 2002 and 2008. Further, while research indicates that people from different socioeconomic status (SES) groups are differentially impacted by tobacco marketing in general [17,18], it is not known whether marketing bans affect different SES groups differently. Therefore, the present study examines marketing awareness between different SES groups within each of the four countries. Overall, this paper addressed three questions: (1) What were the associations between marketing regulations and reported awareness of tobacco advertisements, sponsorships, and promotions, both initially and long-term? (2) Did the associations between marketing ban enactment and reported awareness differ between SES groups? and (3) In which countries and through which specific channels is increased regulation of tobacco marketing still needed?

## 2. Methods

### 2.1. Participants

Participants were adult smokers (aged 18 and older) who were interviewed as part of the ITC-4. The ITC-4 is an ongoing prospective cohort survey conducted with nationally representative respondents from the United Kingdom, Canada, Australia, and the United States. All data collection methods and measurements were standardized across the four countries. The ITC-4 survey has been described in detail elsewhere [19–21]. Briefly, random digit dialing was initially used to recruit current smokers within strata defined by geographic region and community size. Respondents who agreed to participate were typically contacted within one week of recruitment, at which time they completed a 35-minute survey (over the telephone) designed to evaluate the psychosocial and behavioral impact of various national-level tobacco control policies.

The first wave of data collection began in 2002 and has continued annually. Data used in the present study were collected from 2002 through 2008 (Wave 7). Respondents lost to attrition were replenished at each wave using the same recruitment protocols as were used in Wave 1. All respondents who participated in at least one of the seven survey waves were included in the present study, giving a total of 21,615 individuals (5251 in the UK, 5265 in Canada, 4806 in Australia, and 6293 in the US).

### 2.2. Measures

#### 2.2.1. Awareness of Tobacco Marketing Through Specific Channels

Dichotomous data were collected about awareness of tobacco marketing through 15 different channels. The general term “channels” is used to refer to the places where tobacco products are advertised and the means through which they are promoted. Specifically, participants were asked the following stem question: “In the last 6 months, have you noticed cigarettes or tobacco products being advertised in any of the following places?” Channels queried included stores (point-of-sale display awareness, in particular, was only queried in the later survey waves and was not specifically assessed in the present study), billboards, and newspapers/magazines. Participants were also asked if they had seen or heard about any “sport or sporting event” or “music, theatre, art, or fashion event” sponsored by either a cigarette brand or a tobacco company. Respondents were also asked about their awareness of various tobacco promotions using the following item: “In the last 6 months, have you noticed any of the following types of tobacco promotion?” Channels included special price offers, free samples, free gifts/special discount offers on other products, email messages, Internet sites, direct mailings, signs/posters/branded items in bars/pubs/clubs, clothing/other items with a cigarette brand name/logo, leaflets promoting cigarettes or tobacco products, and competitions linked to cigarettes. For each channel, participants were asked to respond “yes” (coded as 1) or “no” (coded as 0). Data on tobacco marketing through four other channels (*i.e.*, TV, radio, movies, and mobile phones) were also collected but were excluded from analyses due to low reported awareness at baseline and throughout the study period.

#### 2.2.2. Overall Awareness of Tobacco Marketing

Two measures were used to assess overall awareness of tobacco marketing: (1) *Salience of pro smoking* was assessed with the following item: “Thinking about everything that happens around you, in the last 6 months, how often have you noticed things that promote smoking?” Participants were given the following response options: never, rarely, sometimes, often, and very often. These responses were dichotomized to indicate whether tobacco marketing was salient (*i.e.*, participants noticed things that promote smoking often or very often) or was not salient (*i.e.*, participants noticed things that promote smoking never, rarely, or sometimes); and (2) *Sum of channels* indicated the total number of channels through which marketing was noticed.

Also, the individual channels were grouped and analyzed according to type. Groups included *advertisements* (*i.e.*, in stores, on billboards, or in newspapers/magazines), *sponsorships* (*i.e.*, sports, arts, or competitions), *price promotions* (*i.e.*, special price offers, free samples, or gifts/discounts), *electronic promotions* (*i.e.*, email messages or Internet sites), and *other promotions* (*i.e.*, direct mailings, signs/posters, branded items, or leaflets). If respondents noticed marketing through any of the channels that comprised a group, then a code of 1 was assigned for that group. If marketing was not noticed through any of the channels that comprised a group, then a code of 0 was assigned.

#### 2.2.3. Sociodemographics and Smoking Behavior

Demographic variables included gender, age at recruitment (measured on a continuous scale), and identified majority/minority group, which was based on the primary means of identifying minorities in each country (*i.e.*, racial/ethnic group in the UK, Canada, and the US, and English language spoken at home in Australia). The heaviness of smoking index (HSI), created in the manner described by Hyland *et al.* [22], was used as a measure of nicotine dependence.

Respondents were also asked about two socioeconomic indicators: annual household income and level of education. Income was grouped into low (less than US$ 30,000 (or £ 30,000 in the UK)), moderate (between US$ 30,000 and US$ 59,999 (or £ 30,000 and £ 44,999 in the UK)), and high categories (equal to or greater than US$ 60,000 (or £ 45,000 in the UK)). Level of education was also grouped into low (completed high school or less in Canada, US, and Australia, or secondary/vocational or less in the UK), moderate (community college/trade/technical school/some university (no degree) in Canada and the US, college/university (no degree) in the UK, or technical/trade/some university (no degree) in Australia), and high categories (completed university or postgraduate in all countries). The income and education responses were combined to create a three-category indicator of SES using the following criteria: if both income and education were low, then SES was defined as low, if either income or education was low, then SES was defined as moderate, and if neither income nor education were low, then SES was defined as high. Respondents who answered only one of the two items were included in the SES category called for by the answered item. Respondents who did not answer either item were excluded from SES-specific analyses.

### 2.3. Statistical Analyses

All analyses were conducted using Stata Version 11 [23]. The generalized estimating equations (GEE) approach was used to determine the extent to which awareness of tobacco marketing changed over time, while accounting for the correlated nature of data within persons across survey waves. Additionally, this approach allowed for the evaluation of population averaged trends across the entire study period without requiring individuals to be present at each survey wave. All GEE models included a specification for the unstructured within-group correlation structure (where a “group” is a person with multiple records), and parameter estimates were computed using robust variance. Models used to estimate dichotomous outcomes included a specification for the binomial distribution of the dependent variable, while models used to estimate continuous outcomes specified the Gaussian distribution for the dependent variable. All multivariate analyses included an adjustment for respondents’ age, gender, minority group, and the heaviness of smoking index. Since quitting smoking may affect awareness of tobacco marketing, participants who quit during the course of the study period did not continue to contribute to the analyses.

Changes in reported tobacco marketing awareness across the entire study period (absolute change, as well as average change per year), and between all consecutive waves (for each individual channel and for the overall awareness measures) were determined for each of the four countries separately. That is, time was the primary predictor of interest in each statistical model. Specifically, absolute changes between the beginning and the end of the study period were assessed with the inclusion of time as a dummy-coded variable in the models, while average changes across the entire study period were assessed with the inclusion of time as a continuous variable in the models. Changes between consecutive waves were assessed with the inclusion of time as dummy-coded categorical variables in the models. Each wave was designated as the reference group until all wave-to-wave comparisons were made. Next, to specifically examine the UK Tobacco Advertising and Promotion Act, differences in awareness change between the UK and the other three countries were assessed by including country-by-change over time interaction terms in all statistical models.

In order to determine whether tobacco marketing regulations had differential awareness change associations among different SES groups, several analyses were conducted separately for the low, moderate, and high SES groups within each country. First, overall change in awareness across the entire study period was estimated for each SES group within each country (*i.e.*, the continuous time variable was the predictor of interest in these analyses). Each statistical model was reanalyzed with the inclusion of SES-by-change over time interaction terms to statistically compare the awareness change experienced by the low SES group to the change experienced by the moderate and high SES groups. For brevity, only those SES differences that were significant are presented in the Results Section. In addition, several other selected analyses were conducted for each SES group based on specific policy changes that occurred in the four countries between 2002 and 2008 (as indicated in Table 1).

## 3. Results

### 3.1. Changes in Reported Exposure to Tobacco Product Marketing

The percentages of respondents in each country who reported being aware of tobacco marketing through each channel at the beginning (2002) and end (2008) of the study period, along with the corresponding odds of absolute change in awareness across the entire study period are presented in Table 2. Significant differences in odds ratios between countries are indicated with asterisks (comparing each country to the UK).

Tables 3–6 present the odds of tobacco marketing awareness change between all consecutive waves within each of the four counties, along with the *per wave* odds of change in awareness across the entire study period. Significant differences in odds ratios between the UK and each of the other three countries are indicated with asterisks in Tables 4–6.

#### 3.1.1. United Kingdom

In the UK, there were significant decreases in reported awareness of tobacco marketing through 13 of the 15 individual channels between 2002 and 2008 with the exception of email messages and Internet sites promoting tobacco products, channels where awareness was low and unchanged throughout the study period (Table 2). At last measurement (either in 2007 or 2008), fewer than 10% of respondents reported being exposed to tobacco marketing through 12 of the 15 channels, with exceptions being awareness of store advertisements (29% aware), special price offers (25%) and sports sponsorships (17%). Overall salience of tobacco marketing decreased significantly from the beginning of the study period (when 20% of respondents reported noticing pro-smoking cues often or very often) to the end of the study period (when only 5% of respondents reported noticing pro-smoking cues often or very often).

The period of time during which the greatest awareness declines occurred in the UK was between 2002 and 2003 (Table 3), which was when the majority of the UK marketing regulations went into effect. Subsequent wave-to-wave comparisons suggest that the effects of marketing restrictions were largely immediate, followed by continuous awareness reduction at a slower rate over time. For example, the steep decline in awareness of newspaper/magazine advertisements between 2002 and 2003 (OR = 0.43, p < 0.001) was followed by modest awareness reductions in the ensuing years (ranging from OR = 0.56 to OR = 0.90).

#### 3.1.2. Canada

Respondents in Canada reported significant decreases in their awareness of tobacco marketing through 11 of the 15 individual channels during the course of the study period (Table 2). These decreases tended to be smaller than those observed in the UK, with the exception of arts sponsorships. At last measurement (in 2008), fewer than 10% of respondents in Canada reported being exposed to tobacco marketing through 11 of the 15 channels, with exceptions being awareness of store advertisements (36% aware—note that Table 2 indicates the percentage measured in 2007 to be consistent with the last measurement in the UK), special price offers (19%), newspaper/magazine advertisements (15%), and sports sponsorships (10%). Similar to the UK, overall salience of tobacco marketing in Canada decreased significantly during the course of the study period, with 20% of respondents reporting tobacco marketing as being salient at the start of the study and only 6% reporting marketing salience at the end of the study.

The greatest awareness declines in Canada occurred between 2007 and 2008 in the following channels: store advertisements, sign/poster promotions, special price offers, and branded item promotions (Table 4). These declines were greater in Canada than they were in the UK during the same period of time (although the store advertisement awareness comparison was not tested).

#### 3.1.3. Australia

Relative to the other three countries, respondents in Australia reported less awareness of tobacco marketing at baseline, which is expected given that many complete marketing bans were already in place in Australia prior to 2002. Awareness did decline modestly over the course of the study period, with significant change occurring in 12 of the 15 individual channels (Table 2). In general, the declines in Australia were not as large as were those observed in the UK, with the exception of arts sponsorships. At last measurement (either in 2007 or 2008), the percentages of respondents aware of tobacco marketing in Australia were reduced to less than 10% in 12 of the 15 channels measured. Exceptions included awareness of store advertisements (26%), sports sponsorships (15%) and special price offers (15%). Similar to the UK and Canada, overall salience of tobacco marketing decreased significantly during the study period, with 15% of respondents reporting pro-smoking salience in 2002, and only 5% reporting salience in 2008. Unlike in the UK and Canada, there were no periods of time during which substantial awareness declines occurred in Australia (Table 5).

#### 3.1.4. United States

In general, respondents in the United States reported more awareness of tobacco marketing at the beginning of the study period and less reduction in awareness across the study period than did respondents in the other three countries (Table 2). There were decreases in awareness through 11 of the 15 individual channels in the US, but these decreases were significantly smaller than were those observed in the UK (with some exceptions). Unlike in the other three countries, the percentages of smokers in the US who still reported being aware of tobacco marketing in 2008 were high. There were only four channels where fewer than 10% of respondents noticed tobacco marketing (*i.e.*, arts and competition sponsorships, email messages and Internet site promotions), and awareness through these channels was relatively low at the start of the study as well. Store advertisements and special price offers, in particular, still reached relatively large percentages of smokers in 2008 (86% and 71%, respectively—note that the 2007 store advertisement percentage is reported in Table 2 to be consistent with the last measurement of this channel in the UK). While overall salience of tobacco marketing did decline in the US, the percentage of respondents who reported tobacco marketing as being salient at last measurement (13%) was more than double the percentages reported in the other three countries (Table 2). Similar to Australia, there were no periods of time during which substantial awareness declines occurred in the US (Table 6).

### 3.2. Changes in Reported Exposure to Tobacco Product Marketing by SES

Changes in awareness across the entire study period (in per wave units) were estimated for each SES group within each country, and differences in changes between SES groups were statistically compared. In general, awareness change tended to be similar among different SES groups. Table 7 indicates the channels through which change in reported awareness differed between SES groups. Out of the 68 possible SES differences (*i.e.*, 15 individual channels plus two overall awareness measurements for each country), there were only 10 statistically significant SES differences in awareness change at alpha = 0.01. After using a Bonferroni adjustment for multiple testing, however, only five SES differences reached significance (*i.e.*, p < 0.001). Specifically, in each of the four countries, the high SES groups experienced greater reductions in the total number of channels through which they reported being aware of tobacco marketing compared to the low SES groups. However, at baseline, the high SES groups in each country were exposed to more marketing channels than were the low SES groups, leaving the high groups more room to experience reduction across the study period. Therefore, the significant SES group differences should not be interpreted as an indicator that marketing regulations had differential impacts on different SES groups. Indeed, at the end of the study period, the total numbers of channels through which respondents reported being aware of tobacco marketing were statistically indistinguishable between SES groups within countries (data not shown).

Reported changes in awareness of different types of tobacco marketing between the survey waves immediately before and after regulations went into effect (as indicated by Table 1) were also estimated for each SES group within each country. Again, awareness changes tended to be similar among different SES groups. There were only two instances where those in the high SES group experienced a greater reduction in awareness relative to those in the low SES group. These exceptions were awareness of billboard advertisements and arts sponsorships in the UK immediately following the enactment of TAPA (data not shown). There were no other SES differences in awareness change immediately following ban enactments.

## 4. Discussion

Exposure to pro-smoking cues promotes the initiation, continuation, and reuptake of cigarette smoking, and the results of this study indicate that tobacco marketing regulations were associated with significant reductions in smokers’ reported awareness of pro-smoking cues. In general, the observed declines in tobacco marketing awareness were greatest immediately following the enactment of regulations, with awareness reduction occurring more slowly in subsequent years. The reported changes in awareness of tobacco marketing were generally similar across different socioeconomic strata, with the exception of billboard advertising and arts sponsorships, which were reduced more sharply among those in the high SES group relative to those in the low SES group.

While the results of this study suggest that, by and large, tobacco marketing regulations are associated with reduced exposure to pro-smoking cues among all SES groups, evidence indicates that certain channels are still being used by tobacco companies to reach significant percentages of smokers in each country. In the UK, smokers still report substantial exposure to tobacco marketing in stores and through special price offers, which are still permitted. Similarly, the 2008 ITC survey reveals that Canadian smokers are still exposed to special price offers, in-store advertisements, and newspaper/magazine advertisements. In 2009, Canada passed an Amendment to the Tobacco Act to restrict marketing via store advertisements and newspaper/magazine advertisements [24]. Therefore, it might be anticipated that awareness through these channels will decline in subsequent years.

Awareness of tobacco marketing was generally lower in Australia than in any other country at the start of this study. This is consistent with the many marketing regulations that were in place in Australia prior to 2002. However, these results show that there are still some gaps in the restrictions in Australia. Point-of-sale displays of cigarettes and tobacco discounting were still permitted in 2008, and the consequences of these allowances are evident in the relatively high percentages of Australian respondents who reported being aware of store advertisements and special price promotions at the last measurement.

Despite the absence of substantial tobacco marketing regulation change during the course of the study period, respondents in the US did experience modest reductions in their awareness of tobacco marketing. This finding is not unexpected given the tobacco industry’s declining expenditures on marketing in the US since 2003 [25]. The percentages of US respondents who reported being aware of tobacco marketing at the end of the study period, however, were still considerably higher compared to the percentages in the other countries. This underscores the need to enact and enforce stricter regulations, particularly on channels that were not regulated by the government but apparently taken advantage of by the tobacco industry, such as store advertisements and special price offers (approximately three-quarters of US smokers were exposed to marketing through these channels at last measurement). Recently, the US Food and Drug Administration enacted “Regulations Restricting the Sale and Distribution of Cigarettes and Smokeless Tobacco to Protect Children and Adolescents,” effective June 2010 [15]. This legislation is intended to reduce the access to and appeal of cigarettes to adolescents. It is an important step in the effort to combat the tobacco epidemic because it interferes with one of the key mechanisms through which tobacco companies increase cigarette consumption—by increasing the number of new smokers. Future research will be able to assess the impact of these new regulations.

The results reported here should be interpreted in light of this study’s strengths and weaknesses. Major strengths include: (1) use of large, nationally representative samples of smokers from four countries, each with differing levels of tobacco marketing regulations; (2) the longitudinal design, which allowed for changes in awareness within countries over time to be assessed; and (3) the statistical modeling approach employed, which accounted for the correlated nature of data within persons, and included all individuals who were present in any survey wave during the course of the study period (thereby maximizing power). Weaknesses include reliance on self-reported awareness of tobacco marketing as an indicator of exposure. It has been shown, though, that such awareness measurements are sensitive to changes in marketing regulations [16]. Cigarette branded items may be an exception, however, because respondents can continue to possess and notice these items many years after the items are banned from distribution. Indeed, branded items were completely banned in the US prior to the start of this study, yet 23% of US respondents still reported being aware of them at last measurement. Secondly, this study did not consider the extent to which tobacco marketing regulations were implemented in each of the countries, nor were enactment/implementation differences between states/provinces within countries examined. Some countries do have sub-national policies, and these could be individually explored in future research. In particular, a comprehensive evaluation of product display awareness at the sub-national level is needed. Lastly, the impacts of tobacco marketing regulations on potential and former smokers, two groups who are also targeted by tobacco companies, were not evaluated in the present study.

## 5. Conclusions

To summarize, in an effort to decrease the disease burden caused by tobacco, and in accordance with Article 13 of the WHO FCTC, several countries have enacted restrictions on the advertising, sponsorship, and promotion of tobacco products. The longitudinal data from this study show that smokers from all SES groups report significant reductions in their awareness of tobacco marketing immediately following the enactment of marketing regulations. The relatively new restrictions in Canada, the United States, and the UK are promising steps toward the continued reduction of influence that tobacco companies have on smokers. However, additional bans/more stringent implementation of existing bans are especially needed on store advertisements and special price offers, and continuous evaluation of the impacts of such marketing regulations is vital.

## Figures and Tables

**Table 1.** Comparison of the extent of tobacco marketing regulations across the study period (2002–2008) in each country.

	United Kingdom							Canada							Australia							United States						
	02	03	04	05	06	07	08	02	03	04	05	06	07	08	02	03	04	05	06	07	08	02	03	04	05	06	07	08
**Advertisements**																												
Stores	-	O	O	O	O	O	O	O	O	O	O	O	O	X	O	O	O	O	O	O	O	-	-	-	-	-	-	-
Billboards	O	X	X	X	X	X	X	X	X	X	X	X	X	X	X	X	X	X	X	X	X	O	O	O	O	O	O	O
Newspapers/magazines	-	X	X	X	X	X	X	O	O	O	O	O	O	O	X	X	X	X	X	X	X	O	O	O	O	O	O	O
**Sponsorships**																												
Sports	-	O	O	X	X	X	X	O	X	X	X	X	X	X	O	O	O	O	X	X	X	O	O	O	O	O	O	O
Arts	-	X	X	X	X	X	X	O	X	X	X	X	X	X	X	X	X	X	X	X	X	O	O	O	O	O	O	O
Competitions	-	X	X	X	X	X	X	X	X	X	X	X	X	X	X	X	X	X	X	X	X	O	O	O	O	O	O	O
**Price Promotions**																												
Special price offers	-	X	X	X	X	X	X	O	O	O	O	O	O	O	-	-	-	-	-	-	-	-	-	-	-	-	-	-
Free samples	-	X	X	X	X	X	X	X	X	X	X	X	X	X	O	O	O	O	O	O	O	O	O	O	O	O	O	O
Gifts	-	X	X	X	X	X	X	X	X	X	X	X	X	X	O	O	O	O	O	O	O	O	O	O	O	O	O	O
**Electronic Promotions**																												
Email messages	-	X	X	X	X	X	X	O	O	O	O	O	O	O	X	X	X	X	X	X	X	-	-	-	-	-	-	-
Internet sites	O	X	X	X	X	X	X	-	-	-	-	-	-	-	O	O	O	O	O	O	O	-	-	-	-	-	-	-
**Other Promotions**																												
Direct mailings	-	O	O	O	O	O	O	O	O	O	O	O	O	O	O	O	O	O	O	O	O	-	-	-	-	-	-	-
Signs/posters	-	O	O	O	O	O	O	O	O	O	O	O	O	O	X	X	X	X	X	X	X	-	-	-	-	-	-	-
Branded items	-	-	-	-	X	X	X	O	O	O	O	O	O	O	X	X	X	X	X	X	X	X	X	X	X	X	X	X
Leaflets	-	X	X	X	X	X	X	O	O	O	O	O	O	O	X	X	X	X	X	X	X	-	-	-	-	-	-	-

X = complete ban, O = partial ban, - = no ban.

**Table 2.** Awareness of tobacco marketing at the beginning (2002) and end (2008) of the study period, and corresponding odds of awareness change. GEE multivariate logistic regression analyses adjusted for age, gender, minority group, and the heaviness of smoking index. Statistically significant between-country differences in odds ratios are indicated with asterisks (UK is reference country).

	**United Kingdom (N = 5251)**				**Canada (N = 5265)**				**Australia (N = 4806)**				**United States (N = 6293)**			
	% aware		change in awareness 02–08		% aware		change in awareness 02–08		% aware		change in awareness 02–08		% aware		change in awareness 02–08	
	2002	2008	OR	p	2002	2008	OR	p	2002	2008	OR	p	2002	2008	OR	p
**Advertisements^1^**	85	32	0.08	<.001	71	44	0.33**	<.001	61	29	0.26**	<.001	95	89	0.53**	<.001
Stores^1^	69	29	0.18	<.001	54	36	0.50**	<.001	55	26	0.29**	<.001	89	86	0.94**	.490
Billboards	58	9	0.07	<.001	28	9	0.26**	<.001	19	5	0.25**	<.001	53	28	0.42**	<.001
Newspapers/magazines	49	7	0.07	<.001	41	15	0.27**	<.001	16	7	0.42**	<.001	64	30	0.26**	<.001
**Sponsorships^1^**	60	19	0.14	<.001	58	18	0.16	<.001	41	17	0.29**	<.001	47	29	0.50**	<.001
Sports	57	17	0.13	<.001	51	10	0.11	<.001	34	15	0.31**	<.001	35	16	0.37**	<.001
Arts^1^	3	1	0.43	.001	23	5	0.18*	<.001	7	1	0.16*	<.001	10	6	0.63	<.001
Competitions	9	2	0.26	<.001	15	4	0.21	<.001	8	1	0.20	<.001	18	9	0.54	<.001
**Price Promotions^1^**	68	23	0.16	<.001	28	31	1.25**	<.001	41	22	0.48**	<.001	91	74	0.36**	<.001
Special price offers	62	25	0.23	<.001	25	19	0.71**	<.001	34	15	0.38**	<.001	86	71	0.49**	<.001
Free samples^1^	13	2	0.15	<.001	3	2	0.86**	.482	6	2	0.37**	<.001	38	22	0.53**	<.001
Gifts	18	3	0.17	<.001	3	4	1.28**	.219	12	3	0.34*	<.001	34	21	0.59**	<.001
**Electronic Promotions**	4	3	0.86	.422	5	3	0.73	.112	4	2	0.45	.005	15	14	1.00	.967
Email messages	2	1	0.68	.200	3	2	0.74	.235	2	1	0.35	.020	10	9	0.91	.517
Internet sites	2	2	1.07	.760	3	2	0.73	.206	3	1	0.56	.043	11	9	1.01	.958
**Other Promotions^1^**	53	12	0.12	<.001	36	13	0.28	<.001	34	12	0.30**	<.001	80	62	0.48**	<.001
Direct mailings^1^	18	2	0.09	<.001	3	1	0.47**	.004	1	0	0.22	.006	48	42	0.77**	<.001
Signs/posters	32	6	0.14	<.001	30	5	0.11	<.001	27	5	0.15	<.001	49	25	0.41**	<.001
Branded items	17	7	0.37	<.001	11	4	0.29	<.001	13	5	0.38	<.001	38	23	0.60*	<.001
Leaflets^1^	17	2	0.12	<.001	3	1	0.41*	.001	3	1	0.54**	.018	15	18	1.24**	.013
**Overall awareness**																
Salience	20	5	0.23	<.001	20	6	0.18	<.001	15	5	0.30	<.001	27	13	0.37	<.001
Sum of channels^1,2^	4.25	1.06	–3.08	<.001	2.94	1.41	–1.42**	<.001	2.38	1.01	–1.28**	<.001	5.97	4.33	–1.26**	<.001

Odds of absolute awareness change between 2002 and 2008; see Tables 3–6 for corresponding odds of awareness change per wave. There was a significant reduction in absolute awareness of advertisements among Canadian respondents between 2002 and 2008 (*i.e.*, OR = 0.33, p < 0.001), for example, but this reduction was not as great as was the reduction experienced by respondents in the UK (which is indicated by the asterisks beside the Canadian OR).

1 Measurements taken in 2007 rather than 2008;

2 Reporting means and regression coefficients rather than percentages and odds ratios.

* p < 0.01;

** p < 0.001 for between-country comparisons.

**Table 3.** Odds of tobacco marketing awareness change in the **United Kingdom** between consecutive survey waves and across all waves. GEE multivariate logistic regression analyses adjusted for age, gender, minority group, and the heaviness of smoking index. Statistically significant between-country differences in odds ratios are indicated with asterisks in the following 3 tables (comparing UK to the other 3 countries).

	**Consecutive Waves**												**All Waves** (per wave units)	
	**2002–2003**		**2003–2004**		**2004–2005**		**2005–2006**		**2006–2007**		**2007–2008**		**2002–2008**	
	OR	p	OR	p	OR	p	OR	p	OR	p	OR	p	OR	p
**Advertisements^1^**	0.39	<.001	0.87	.024	0.56	<.001	0.64	<.001	0.63	<.001	0.26^2^	<.001	0.58	<.001
Stores^1^	0.68	<.001	0.94	.260	0.62	<.001	0.67	<.001	0.69	<.001	-	-	0.72	<.001
Billboards	0.36	<.001	0.77	<.001	0.61	<.001	0.75	.001	0.48	<.001	1.19	.150	0.61	<.001
Newspapers/magazines	0.43	<.001	0.69	<.001	0.56	<.001	0.72	.001	0.66	<.001	0.90	.421	0.61	<.001
**Sponsorships**	0.66	<.001	0.78	<.001	0.46	<.001	0.71	<.001	0.87	.074	0.88^2^	.123	0.68	<.001
Sports	0.68	<.001	0.77	<.001	0.44	<.001	0.71	<.001	0.87	.092	0.89	.158	0.68	<.001
Arts^1^	0.58	.006	1.11	.652	0.97	.909	0.94	.801	0.73	.283	-	-	0.87	.001
Competitions	0.56	<.001	0.96	.779	0.42	<.001	1.38	.114	0.79	.270	1.06	.771	0.76	<.001
**Price Promotions**	0.46	<.001	0.65	<.001	0.73	<.001	0.94	.350	0.77	<.001	1.18^2^	.017	0.74	<.001
Special price offers	0.52	<.001	0.69	<.001	0.72	<.001	0.94	.360	0.78	<.001	1.24	.002	0.76	<.001
Free samples^1^	0.47	<.001	0.48	<.001	0.63	.011	0.85	.479	1.22	.375	-	-	0.60	<.001
Gifts	0.45	<.001	0.48	<.001	0.88	.447	0.87	.425	0.73	.112	1.39	.106	0.66	<.001
**Electronic Promotions**	1.18	.250	0.93	.658	0.84	.328	0.99	.950	0.95	.765	0.98	.933	0.96	.156
Email messages	1.62	.018	0.68	.069	0.80	.372	0.99	.953	0.85	.536	0.93	.806	0.91	.010
Internet sites	0.80	.235	1.27	.273	0.97	.892	0.96	.845	1.08	.714	1.06	.787	1.01	.662
**Other Promotions**	0.50	<.001	0.69	<.001	0.64	<.001	0.88	.092	0.60	<.001	0.86^2^	.170	0.67	<.001
Direct mailings^1^	0.55	<.001	0.40	<.001	0.51	<.001	1.04	.842	0.72	.142	-	-	0.55	<.001
Signs/posters	0.57	<.001	0.91	.227	0.62	<.001	0.80	.029	0.52	<.001	1.07	.637	0.71	<.001
Branded items	0.59	<.001	0.99	.903	0.81	.039	0.76	.025	0.83	.165	1.25	.107	0.82	<.001
Leaflets^1^	0.48	<.001	0.54	<.001	0.70	.023	1.04	.822	0.63	.017	-	-	0.63	<.001
**Overall awareness**														
Salience	0.44	<.001	0.87	.172	0.83	.095	0.90	.367	1.03	.804	0.79	.086	0.78	<.001
Sum of channels^3^	–1.26	<.001	–0.49	<.001	–0.70	<.001	–0.29	<.001	–0.35	<.001	–0.26^2^	<.001	–0.59	<.001

Odds of awareness change across all waves are on the same scale as odds of awareness change between consecutive waves (*i.e.*, in wave units). The significant reduction in awareness of advertisements among respondents in the UK between 2002 and 2003 (*i.e.*, OR = 0.39, p < 0.001), for example, was statistically greater than were the corresponding reductions in the three comparison countries (as indicated with asterisks in the following three tables).

1 Last measured in 2007;

2 Based on fewer channels than in previous years;

3 Reporting means and regression coefficients rather than percentages and odds ratios.

**Table 4.** Odds of tobacco marketing awareness change in **Canada** between consecutive survey waves and across all waves. GEE multivariate logistic regression analyses adjusted for age, gender, minority group, and the heaviness of smoking index. Statistically significant differences in odds ratios between Canada and the UK are indicated with asterisks.

	**Consecutive Waves**												**All Waves** (per wave units)	
	**2002–2003**		**2003–2004**		**2004–2005**		**2005–2006**		**2006–2007**		**2007–2008**		**2002–2008**	
	OR	p	OR	p	OR	p	OR	p	OR	p	OR	p	OR	p
**Advertisements**	0.74**	<.001	1.14*	.031	0.75**	<.001	0.70	<.001	0.75	<.001	0.47**	<.001	0.77**	<.001
Store	0.87*	.009	1.29**	<.001	0.78*	<.001	0.73	<.001	0.78	<.001	0.35	<.001	0.82**	<.001
Billboards	0.83**	.002	0.74	<.001	0.87**	.072	0.90	.213	0.63	<.001	0.86	.184	0.80**	<.001
Newspapers/magazines	0.85**	.003	0.76	<.001	0.92**	.170	0.67	<.001	0.73	<.001	0.93	.443	0.79**	<.001
**Sponsorship**	0.66	<.001	0.76	<.001	0.64**	<.001	0.67	<.001	0.73	<.001	0.70	<.001	0.69	<.001
Sports	0.69	<.001	0.79	<.001	0.61**	<.001	0.66	<.001	0.68	<.001	0.72	.002	0.69	<.001
Arts	0.67	<.001	0.68	<.001	0.67	<.001	0.90	.375	0.65	.002	0.77	.137	0.71**	<.001
Competitions	0.80	.007	0.67	<.001	0.86**	.189	0.88	.300	0.82	.134	0.63	.014	0.79	<.001
**Price Promotions**	1.28**	<.001	1.59**	<.001	0.84	.002	0.85	.009	0.86	.013	0.55^2^**	<.001	0.98**	.032
Special price offers	1.37**	<.001	1.61**	<.001	0.83	.001	0.87	.020	0.81	.001	0.56**	<.001	0.98**	.068
Free samples^1^	0.66	.033	1.07*	.767	0.71	.137	1.31	.323	1.32	.233	-	-	0.96**	.335
Gifts	1.26**	.130	0.99*	.958	1.00	.982	1.17	.354	0.92	.617	0.94	.777	1.04**	.085
**Electronic Promotions**	1.53	<.001	0.93	.534	0.65	.001	0.97	.829	1.02	.882	0.79	.220	0.93	.003
Email messages	1.68	.001	1.05	.726	0.54	<.001	0.98	.934	0.85	.436	0.93	.780	0.92	.002
Internet sites	1.59*	.001	0.71	.017	0.71	.063	1.00	.999	1.38	.095	0.65	.075	0.92	.003
**Other Promotions**	0.71**	<.001	0.84	.008	0.81	.003	0.83	.017	0.69	<.001	0.48^2^*	<.001	0.77**	<.001
Direct mailings^1^	0.82	.272	0.88*	.565	0.50	.011	1.35	.279	0.97	.906	-	-	0.84**	<.001
Signs/posters	0.71	<.001	0.79	.001	0.81	.009	0.74	.001	0.76	.009	0.44**	<.001	0.75*	<.001
Branded items	0.97**	.700	0.85	.098	0.84	.117	1.08	.543	0.68	.003	0.58*	.006	0.86	<.001
Leaflets^1^	0.93*	.702	0.63	.051	1.03	.899	0.99	.971	0.68	.185	-	-	0.84**	<.001
**Overall awareness**														
Salience	0.68**	<.001	0.65	<.001	0.76	.009	0.97	.762	1.03	.776	0.52	<.001	0.77	<.001
Sum of channels^3^	–0.24**	<.001	–0.18**	<.001	–0.36**	<.001	–0.32	<.001	–0.32	<.001	–0.49^2^**	<.001	–0.30**	<.001

Odds of awareness change across all waves are on the same scale as odds of awareness change between consecutive waves (*i.e.*, in wave units).

1 Last measured in 2007;

2 Based on fewer channels than in previous years;

3 Reporting means and regression coefficients rather than percentages and odds ratios.

* p < 0.01;

** p < 0.001 for between-country comparisons.

**Table 5.** Odds of tobacco marketing awareness change in **Australia** between consecutive survey waves and across all waves. GEE multivariate logistic regression analyses adjusted for age, gender, minority group, and the heaviness of smoking index. Statistically significant differences in odds ratios between Australia and the UK are indicated with asterisks.

	**Consecutive Waves**												**All Waves** (per wave units)	
	**2002–2003**		**2003–2004**		**2004–2005**		**2005–2006**		**2006–2007**		**2007–2008**		**2002–2008**	
	OR	p	OR	p	OR	p	OR	p	OR	p	OR	p	OR	p
**Advertisements^1^**	0.71**	<.001	0.87	.010	0.73*	<.001	0.83*	.001	0.69	<.001	0.24^2^	<.001	0.72**	<.001
Store^1^	0.72	<.001	0.94	.261	0.72	<.001	0.82	.002	0.73	<.001	-	-	0.79**	<.001
Billboards	0.67**	<.001	0.92	.312	0.77	.005	0.94	.574	0.70	.001	0.79	.075	0.80**	<.001
Newspapers/magazines	0.86**	.041	0.64	<.001	0.91**	.331	1.07*	.514	0.62	<.001	1.29	.053	0.84**	<.001
**Sponsorship**	0.76	<.001	0.88	.034	0.77**	<.001	0.73	<.001	0.78	.001	0.82^2^	.020	0.79**	<.001
Sports	0.89**	.033	0.93	.199	0.77**	<.001	0.73	<.001	0.77	.001	0.86	.080	0.82**	<.001
Arts^1^	0.47	<.001	0.80	.170	0.66	.035	1.05	.835	0.60	.052	-	-	0.70**	<.001
Competitions	0.59	<.001	0.53*	<.001	0.86*	.498	0.92	.709	0.92	.704	0.88	.654	0.72	<.001
**Price Promotions**	0.82**	<.001	0.85*	.003	0.94*	.324	0.82	.002	0.89	.078	0.71^2^**	<.001	0.85**	<.001
Special price offers	0.85**	.001	0.90*	.070	0.94*	.282	0.80	.001	0.93	.298	0.71**	<.001	0.87**	<.001
Free samples^1^	0.59	<.001	0.62	.004	0.80	.294	1.22	.339	1.03	.887	-	-	0.78**	<.001
Gifts	0.85**	.063	0.75*	.004	0.91	.361	0.86	.229	0.87	.332	0.79	.159	0.84**	<.001
**Electronic Promotions**	1.16	.265	1.31	.049	0.81	.137	0.73	.061	1.05	.795	0.48	.005	0.92	.004
Email messages	1.60	.007	0.98	.906	0.91	.599	0.60	.022	0.79	.399	0.52	.165	0.87	<.001
Internet sites	0.80	.223	1.74	.002	0.83	.285	0.77	.201	1.14	.532	0.54	.021	0.97	.366
**Other Promotions**	0.67**	<.001	0.86	.020	0.80	.001	0.79	.004	0.84	.045	0.63^2^	<.001	0.78**	<.001
Direct mailings^1^	0.62	.106	1.10*	.751	0.61	.244	1.08	.883	0.48	.231	-	-	0.79**	<.001
Signs/posters	0.64	<.001	0.83	.008	0.76	<.001	0.83	.048	0.79*	.023	0.55*	<.001	0.76*	<.001
Branded items	0.77	.001	0.85	.087	0.95	.658	0.73	.009	0.95	.670	0.87	.338	0.85	<.001
Leaflets^1^	0.45	<.001	0.87	.654	1.36	.294	1.00	.997	1.02	.950	-	-	0.88**	.025
**Overall awareness**														
Salience	0.63*	<.001	0.69	<.001	0.98	.864	1.07	.550	1.11	.362	0.60	<.001	0.85**	<.001
Sum of channels^3^	–0.44**	<.001	–0.18**	<.001	–0.25**	<.001	–0.20	<.001	–0.21*	<.001	–0.44^2^*	<.001	–0.27**	<.001

Odds of awareness change across all waves are on the same scale as odds of awareness change between consecutive waves (*i.e.*, in wave units).

1 Last measured in 2007;

2 Based on fewer channels than in previous years;

3 Reporting means and regression coefficients rather than percentages and odds ratios.

* p < 0.01;

** p < 0.001 for between-country comparisons.

**Table 6.** Odds of tobacco marketing awareness change in the **United States** between consecutive survey waves and across all waves. GEE multivariate logistic regression analyses adjusted for age, gender, minority group, and the heaviness of smoking index. Statistically significant differences in odds ratios between the US and the UK are indicated with asterisks.

	**Consecutive Waves**												**All Waves** (per wave units)	
	**2002–2003**		**2003–2004**		**2004–2005**		**2005–2006**		**2006–2007**		**2007–2008**		**2002–2008**	
	OR	p	OR	p	OR	p	OR	p	OR	p	OR	p		_
**Advertisements**	0.78**	.031	0.82	.051	0.77*	.006	0.93*	.411	1.18**	.076	0.55**	<.001	0.85**	<.001
Store	0.95*	.577	0.89	.174	0.90**	.189	0.97**	.743	1.26**	.004	0.50	<.001	0.92**	<.001
Billboards	0.79**	<.001	0.93	.246	0.87**	.023	0.91	.107	0.74**	<.001	0.98	.777	0.86**	<.001
Newspapers/magazines	0.88**	.023	0.93*	.186	0.76*	<.001	0.70	<.001	0.73	<.001	0.82	.005	0.79**	<.001
**Sponsorship**	1.12**	.041	0.66	<.001	0.82**	.001	0.87	.027	0.95	.429	0.69	<.001	0.84**	<.001
Sports	1.24**	<.001	0.59*	<.001	0.86**	.023	0.85	.023	0.95	.502	0.73	<.001	0.84**	<.001
Arts	0.76	.008	1.04	.759	0.99	.954	1.06	.630	0.76	.037	0.61	.006	0.92	<.001
Competitions	0.94**	.446	0.83	.028	0.80*	.020	0.96	.671	1.28	.008	0.69	.001	0.92**	<.001
**Price Promotions**	0.55	<.001	0.83	.017	0.80	.004	0.91	.203	1.07*	.316	1.00	.946	0.87**	<.001
Special price offers	0.64	<.001	0.71	<.001	0.86	.030	0.92	.23	1.17**	.023	1.16	.038	0.89**	<.001
Free samples	0.71**	<.001	0.81*	.001	0.84	.016	0.95	.523	1.14	.071	0.53	<.001	0.85**	<.001
Gifts	0.87**	.030	1.01**	.857	1.00	.984	1.07	.291	0.86	.034	0.73*	<.001	0.95**	<.001
**Electronic Promotions**	1.33	<.001	1.28	.001	0.86	.035	0.95	.515	1.17	.051	0.61	<.001	1.04	.008
Email messages	1.41	<.001	1.27*	.003	0.87	.094	0.92	.344	1.18	.068	0.54	<.001	1.03*	.053
Internet sites	1.23	.020	1.26	.011	0.78	.006	1.00	.988	1.19	.085	0.70	.003	1.03	.095
**Other Promotions**	0.71**	<.001	1.08**	.247	0.86*	.018	0.76	<.001	0.96**	.559	0.90	.094	0.87**	<.001
Direct mailings	0.86**	.003	1.33**	<.001	0.95*	.322	0.79	<.001	0.90	.062	0.93	.242	0.95**	<.001
Signs/posters	0.78*	<.001	0.69*	<.001	1.08**	.220	0.78	<.001	1.16**	.042	0.80	.004	0.87**	<.001
Branded items	0.80*	<.001	1.00	.951	0.86	.022	0.92	.228	0.93	.324	1.01	.881	0.91**	<.001
Leaflets	1.00**	.993	1.30**	.001	0.97	.719	0.83	.026	1.18*	.045	0.74	.001	1.01**	.407
**Overall awareness**														
Salience	0.82**	.002	0.72	<.001	0.81	.009	1.20	.025	1.03	.724	0.63	<.001	0.88**	<.001
Sum of channels^1^	–0.32**	<.001	–0.28*	<.001	–0.28**	<.001	–0.33	<.001	–0.06*	.404	–0.51**	<.001	–0.28**	<.001

Odds of awareness change across all waves are on the same scale as odds of awareness change between consecutive waves (*i.e.*, in wave units).

1 Reporting means and regression coefficients rather than percentages and odds ratios.

* p < 0.01;

** p < 0.001 for between-country comparisons.

**Table 7.** Statistically significant SES group differences in odds of tobacco marketing awareness change across the entire study period. GEE multivariate logistic regression analyses adjusted for age, gender, minority group, and the heaviness of smoking index. Statistically significant differences between the low SES group and other SES groups are indicated with asterisks.

**Country**	**Channel**	**Low SES**		**Moderate SES**		**High SES**	
		OR	p	OR	p	OR	p
**United Kingdom**							
	Billboards	0.67	<.001	0.59**	<.001	0.57**	<.001
	Sum of channels^1^	–0.53	<.001	–0.61**	<.001	–0.62**	<.001
**Canada**							
	Competitions	0.85	<.001	0.84	<.001	0.73*	<.001
	Sum of channels^1^	–0.24	<.001	–0.29	<.001	–0.34**	<.001
**Australia**							
	Sports	0.85	<.001	0.82	<.001	0.77*	<.001
	Sum of channels^1^	–0.22	<.001	–0.27	<.001	–0.34**	<.001
**United States**							
	Arts	1.02	.657	0.94	.041	0.88*	<.001
	Free samples	0.88	<.001	0.86	<.001	0.82*	<.001
	Email messages	1.13	.001	0.99*	.731	1.02	.283
	Sum of channels^1^	-	-	–0.26*	<.001	–0.34**	<.001

* p < 0.01;

** p < 0.001;

1 Reporting regression coefficients rather than odds ratios.
